# Early long-term administration of the CSF1R inhibitor PLX3397 ablates microglia and reduces accumulation of intraneuronal amyloid, neuritic plaque deposition and pre-fibrillar oligomers in 5XFAD mouse model of Alzheimer’s disease

**DOI:** 10.1186/s13024-018-0244-x

**Published:** 2018-03-01

**Authors:** Justyna Sosna, Stephan Philipp, Ricardo Albay, Jorge Mauricio Reyes-Ruiz, David Baglietto-Vargas, Frank M. LaFerla, Charles G. Glabe

**Affiliations:** 1 Department of Molecular Biology and Biochemistry, University of California, Irvine, USA; 2 Biochemistry Department, Faculty of Science and Experimental Biochemistry Unit, King Fahd Medical Research Center, King Abdulaziz University, Jeddah, 21589 Saudi Arabia; 3 Institute for Memory Impairments and Neurological Disorders (UCIMIND), University of California, Irvine, USA

**Keywords:** Alzheimer’s disease, Amyloid beta, Plaques, Intraneuronal amyloid, Microglia, Neuroinflammation

## Abstract

**Background:**

Besides the two main classical features of amyloid beta aggregation and tau-containing neurofibrillary tangle deposition, neuroinflammation plays an important yet unclear role in the pathophysiology of Alzheimer’s disease (AD). Microglia are believed to be key mediators of neuroinflammation during AD and responsible for the regulation of brain homeostasis by balancing neurotoxicity and neuroprotective events. We have previously reported evidence that neuritic plaques are derived from dead neurons that have accumulated intraneuronal amyloid and further recruit Iba1-positive cells, which play a role in either neuronal demise or neuritic plaque maturation or both.

**Methods:**

To study the impact of microglia on neuritic plaque development, we treated two-month-old 5XFAD mice with a selective colony stimulation factor 1 receptor (CSF1R) inhibitor, PLX3397, for a period of 3 months, resulting in a significant ablation of microglia. Directly after this treatment, we analyzed the amount of intraneuronal amyloid and neuritic plaques and performed behavioral studies including Y-maze, fear conditioning and elevated plus maze.

**Results:**

We found that early long-term PLX3397 administration results in a dramatic reduction of both intraneuronal amyloid as well as neuritic plaque deposition. PLX3397 treated young 5XFAD mice also displayed a significant decrease of soluble fibrillar amyloid oligomers in brain lysates, a depletion of soluble pre-fibrillar oligomers in plasma and an improvement in cognitive function measured by fear conditioning tests.

**Conclusions:**

Our findings demonstrate that CSF1R signaling, either directly on neurons or mediated by microglia, is crucial for the accumulation of intraneuronal amyloid and formation of neuritic plaques, suggesting that these two events are serially linked in a causal pathway leading to neurodegeneration and neuritic plaque formation. CSF1R inhibitors represent potential preventative or therapeutic approach that target the very earliest stages of the formation of intraneuronal amyloid and neuritic plaques.

## Background

AD is classically characterized by the presence of neurofibrillary tangles (NFTs), containing aggregates of the microtubule binding protein tau and plaque deposits composed of aggregates of the amyloid beta peptide (Aβ) [1, 2]. The amyloid cascade hypothesis has evolved over the past 30 years as one of the leading theories of disease, which states that the secretion of Aβ leads to the formation of toxic oligomers that play a causal role in AD [3]. However, the cellular and molecular mechanism by which Aβ aggregates induce these pathological damages and cause dementia remains unclear. Semagacestat, an inhibitor of γ-secretase which creates the carboxyl terminus of Aβ accelerated the decline in cognitive function in clinical trials [4]. We have proposed an alternative formulation of the amyloid hypothesis, based on the observations that FAD PS mutations interfere with the carboxyl-terminal trimming of the initial “long” Aβ cleavage products leading to their aggregation and accumulation in neurons as intraneuronal amyloid [5]. Several transgenic animal models of Aβ deposition that contain FAD PS mutations demonstrate robust intraneuronal amyloid aggregates that occur prior to plaque deposition and in human brain is associated with early stages of AD [6, 7]. The intraneuronal amyloid is aggregated in an amyloid-like structure because it reacts with aggregation-specific antibodies and or thioflavin S [5, 6, 8]. We hypothesized that the intraneuronal amyloid forms the core of the neuritic plaque after the death of the neuron [5]. The evidence for this includes the observation that the core of neuritic plaques contains the same intraneuronal aggregated amyloid immunoreactivity and stains with dyes specific for DNA [5]. Some of the neuritic plaques also contain NeuN immunoreactivity, providing further evidence of their neuronal origin [5]. Since neuritic plaques are typically surrounded by a halo of microglia, we also have hypothesized that neuritic plaques and cored plaques are developmentally related with microglia removing neuronal debris to produce cored plaques [5]. In order to test critical aspects of these hypotheses, we examined the effect of early ablation of microglia in the 5XFAD mouse model that exhibits widespread and robust intraneuronal amyloid immunoreactivity beginning at 1.5 months, prior to neuritic plaque deposition that begins at 2 months and is robust at 5 months [6]. We pharmacologically ablated microglia with PLX3397, a previously described orally-administered selective CSF1R/c-kit inhibitor that is able to cross the blood-brain barrier [9, 10]. Previous studies have determined the optimal dosing and the time-window for PLX3397-induced microglial ablation and it was reported that a 2 month treatment with PLX3397 was reversible in a short period of 3–14 days and had no effect on cognition and behavior of 5xFAD mice [9]. Microglia repopulation was shown to depend on the internal pool of remaining microglia, which in turn is dependent on IL-1 receptor signaling [11]. In contrast to previous studies starting treatment in ten-month-old 5XFAD mice with no effect on Aβ levels or plaque load [10], we started PLX3397 treatment in mice with an age of 2 months. We found that this early treatment with PLX3397 profoundly inhibited not only neuritic plaque formation but also intraneuronal amyloid accumulation. This observation indicates that CSF1R signaling or the presence of microglia is necessary for neuritic plaque formation and that intraneuronal amyloid accumulation and neuritic plaque formation are developmentally linked in a causal series with intraneuronal amyloid upstream of neuritic plaques, consistent with the hypothesis that intraneuronal amyloid is the penultimate source for the amyloid in the center of neuritic plaques.

## Methods

### Animal studies

All mouse experiments were performed according to animal protocols approved by the Institutional Animal Care and Use Committee at the University of California, Irvine. The 5XFAD (B6SJL-Tg(APPSwFlLon,PSEN1*M146 L*L286 V)6799Vas/Mmjax) mice were obtained from The Jackson Laboratory [6]. Mice were housed in groups of 2 to 5 or single-housed for aggressive males, under a 12-h light/12-h dark cycle at 21 °C, with food and water ad libitum. All transgenic animals were genotyped by qPCR (automated genotyping using human APP and PS1 primers, TransnetYX, Cordova, TN) and only animals expressing comparable levels of the human APP and PS1 were chosen for the study. To pharmacologically ablate microglia, six males and nine females of two-month-old 5XFAD mice were treated with PLX3397 (290 mg/kg formulated in standard chow) [9, 10]. Age-matched control groups, ten males and eight females, were fed same standard chow without PLX3397. Previous studies have tested the effects of PLX3397 on wild-type animals and performed the necessary controls [9, 10]. Wild-type littermates were fed with the same PLX3397 chow (two males and two females) or without drug (three males and three females). After 3 months of controlled chow administration, the behavior of treated mice (12 5XFAD animals (five males and seven females) with drug and 11 5XFAD animals (six males and five females) without drug) as well as age-matched controls was analyzed prior to euthanasia. At 5 months, 5XFAD mice display extensive amyloid pathology, manifested predominantly by the presence of intraneuronal amyloid and neuritic plaque accumulation. After behavioral testing, mice were euthanized, blood samples collected intracardiacally with a 21 G needle in a 1 mL syringe and mixed with 0.1 mM EDTA solution to prevent clotting. Each blood sample was centrifuged for 15 min at 2500×g to obtain the plasma fraction. Further, mice were intracardially perfused using a peristaltic pump with approximately 120 ml of phosphate buffered saline over 10 min. Brains were isolated and one hemisphere was fixed in 4% (*w*/*v*) paraformaldehyde while the other half was microdissected to isolate cortex and hippocampus and snap frozen in liquid nitrogen for further analysis.

### Immunohistochemistry and confocal microscopy

Paraformaldehyde-fixed brain tissues from 5XFAD mice and age-matched controls were sectioned with a Vibratome Series 1000 vibrating microtome (The Vibratome Co., St. Louis, section thickness 50 μm). Free-floating sections were collected in PBS containing 0.02% (*w*/*v*) NaN_3_, pH 7.4 and stored at 4 °C prior to staining. Sections were washed with PHEM buffer (60 mM Pipes, 25 mM Hepes, 10 mM EGTA, 2 mM MgCl_2_ pH 6.9), permeabilized in 0.1% (*v*/v) Triton X-100 in PHEM buffer for 30 min and blocked by 1 h incubation in PHEM containing 2% (*w*/*v*) BSA, 1.5% (v/v) goat serum and mouse-on-mouse polymer basic kit according to manufacturer’s protocol (BMK-2202, Vectashield) in order to block non-specific binding to endogenous mouse IgG in the mouse tissue.

Tissue slices were probed with primary antibodies against Iba1 (ionized calcium-binding adapter molecule 1) (1 μg / ml of 019–19,741, Wako Pure Chemicals) in combination with purified anti-β-amyloid, 1–16 antibody (5 μg / ml of 6E10, 803,003, BioLegend) in PHEM buffer overnight at room temperature. After rinsing 3 times with PHEM buffer, sections were blocked in PHEM with 2% (*w*/*v*) BSA for 1 h and incubated with highly cross-absorbed goat-anti-rabbit or goat-anti-mouse, respectively, secondary antibodies (1:250) coupled to Alexa Fluor 488 or 647 dyes (A-11034 or A32728, Invitrogen, Thermo Fisher Scientific). Total Iba1 positive cells, the area fraction of plaques and intraneuronal amyloid were quantified by imaging whole sections of comparable regions of the brain from each animal with a Leica TCS SP8 confocal microscope using a 63× objective. Neuritic plaques were determined by a presence of a diffuse DAPI-positive core surrounded by 6E10-positive dystrophic neurites [5]. Immunofluorescence data were analyzed by Volocity 6.3 high-performance 3D imaging software (PerkinElmer) in which objects were counted automatically based on the threshold of cellular size and intensity. Quantification was followed by statistical analyses applying two-tailed unpaired t-test or *one*-*way ANOVA* with post-hoc *Tukey* HSD (Honestly Significant Difference) by IBM SPSS Statistics Version 24 software. Data are shown as mean ± SEM. *p* values are as follows: *p < 0.05, **p < 0.01, ***p < 0.001 ****p < 0.0001, *****p < 0.00001 and ******p < 0.000001.

### Dot blot analyses

The flash-frozen cortex and hippocampus from each brain hemisphere were ground with a bullet blender and lysis beads (Pink, Next Advance, Eppendorf), in TNE total lysis buffer (20 mM Tris (pH 7.5), 150 mM NaCl, 1% (*v*/v) Triton X-100, 1:10 complete protease inhibitors (Roche)). The mixture was centrifuged (20,000×g, 15 min, 4 °C) and the total protein concentration of the supernatant was assessed by the BCA protein assay (Pierce, ThermoScientific). Afterwards, an equal amount of protein (6 μg) was spotted on a nitrocellulose membrane (0.2 μm). Similarly, an equal amount of plasma (1 μg) for each animal was spotted on the nitrocellulose membrane (0.2 μm) and followed the same procedure. Membranes were blocked with 2% (*w*/*v*) non-fat milk for 1 h followed by an overnight incubation at room temperature with respective antibodies: rabbit monoclonal antibody mA11–204 and mA11–118 [8] detecting pre-fibrillar oligomers or rabbit monoclonal antibodies mOC23 and mOC78 [12], detecting fibrillar oligomers. Membranes were washed three times with PBS/0.1% (*v*/v) Triton X-100 and incubated for 1 h at room temperature with goat-anti-rabbit secondary IgG (H + L) coupled to horseradish peroxidase (111–035-144, Jackson ImmunoResearch). Dot blots were incubated with ECL substrate (Clarity Western, 170–5061, Biorad) and the chemiluminescent signal was detected by a digital imaging using a CCD camera. The densitometric analyses of dot blots were performed by the quantitation of total integrated density after automated light background subtraction and uniform adjustment of the background using ImageJ 1.51j8 (Wayne Rasband, NIH).

### Behavioral tests

#### Elevated plus maze

Animals were tested for anxiety-like behavior on an elevated plus maze, based on a previously described protocol [13]. Under normal conditions, rodents enter significantly fewer times into the open arms as well as spend significantly less time in open arms (avoidance of heights/open spaces) when compared to the closed arms (approach toward dark, enclosed spaces). The elevated plus maze consists of 4 intersecting arms (5 × 30 cm), two of which have walls (“closed”, 15 cm in height) and the other two of which have no walls (“open”). The entire maze is elevated 40 cm above the ground and placed in the center of a room with lighting adjusted to 15 lx. Animals were placed at the junction of these arms and allowed to freely explore the maze for 5 min. Video footage was captured and later analyzed by a blinded investigator for the time spent in open and closed arms, and the total number of arm entries. An animal was considered in an arm whenever the body (not including the tail) was entirely in the arm. The maze was thoroughly cleaned with 70% ethanol in between trials.

#### Y-maze

Y-maze spontaneous alternation was conducted to measure the willingness of rodents to explore new environments. Mice typically prefer to explore a new arm/region rather than returning to the region previously visited within the maze [14]. The Y maze consisted of a central arm with two sides arms positioned perpendicular to the main arm. Each arm was 21 cm long and the maze width was 13.5 cm. The walls of the maze were made of transparent acrylic and were 20 cm tall. Each mouse was placed at the end of one arm and allowed to move freely through the maze during an 8-min session. The series of arms entries was recorded visually. An arm entry was considered when the mouse has completely entered in the arm. Alternation was defined as successive entries into the three arms on overlapping triplet sets.

#### Contextual fear conditioning

The fear conditioning test is based on the Pavlovian fear conditioning paradigm, in which a previously neutral (conditioned) stimulus is paired with an aversive (unconditioned) stimulus, which results in a measurable state of fear [15]. Studies have demonstrated that induced fear responses like freezing are dependent on the integrity of the amygdala complex [16]. However, the nature by which a specific stimulus produces fear-eliciting properties remains unclear [17]. During training, mice were placed in the fear-conditioning chamber (San Diego Instruments, San Diego, CA) and allowed to explore for 2 min and 30 s before receiving one electric foot shocks (duration, 2 s; intensity, 0.2 mA). Animals were returned to the home cage 30 s after the foot shock. Twenty-four hours later, behavior in the conditioning chamber was video recorded for 5 min and subsequently analyzed for freezing, which was defined as the absence of all movement except for respiration.

## Results

### Early, long-term administration of PLX3397 inhibits the accumulation of intraneuronal amyloid and formation of neuritic plaques

**Fig. 1:**
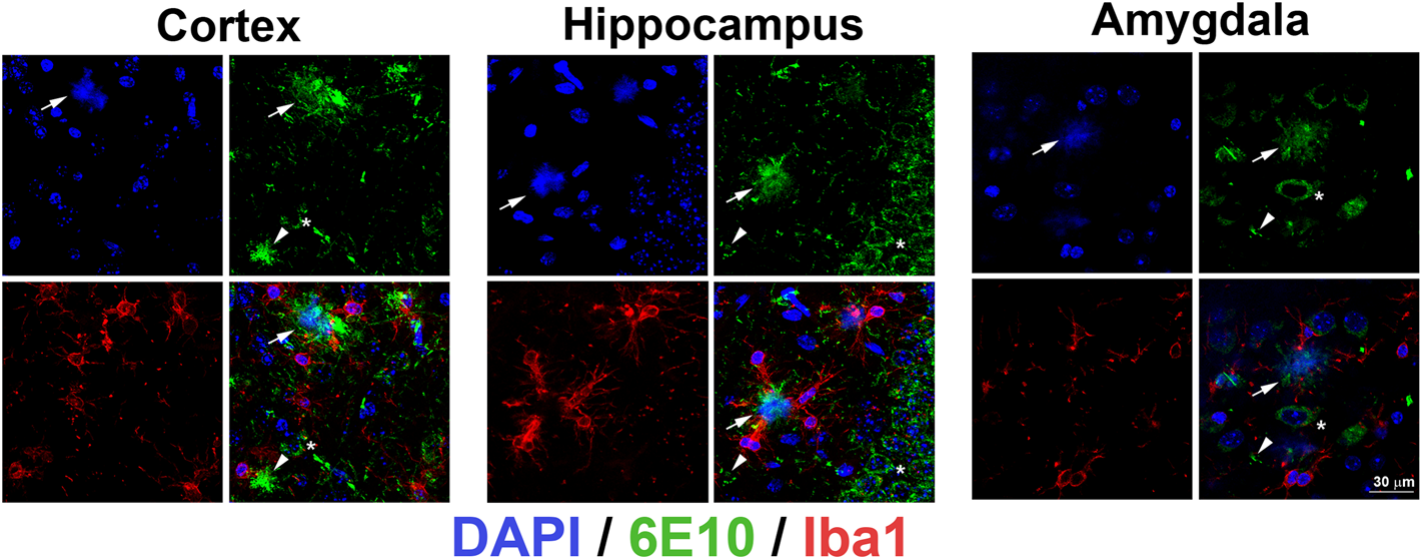
Intraneuronal amyloid and neuritic plaques are present in 5 mo 5XFAD mice. Representative 63× images of intraneuronal amyloid (asterisk), neuritic plaques (arrows) with DAPI-positive core (blue) surrounded by a 6E10-positive amyloid rim (green) and Iba1-positive cells (red) in cortex, hippocampus and amygdala. Arrowheads point to non-neuritic 6E10-positive plaques without a DAPI-positive center. The confocal acquisition was applied and a total z-plane of 10 μm was merged into single overlay images. Bar = 30 μm

**Fig. 2:**
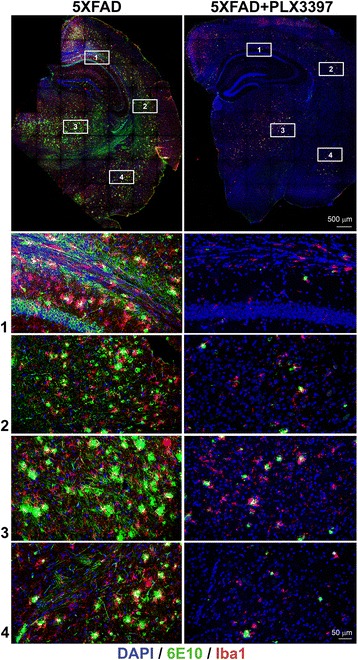
Long-term administration of PLX3397 ablates microglia and reduces neuritic plaque formation in 5XFAD mice. Top panels: representative 20× images of coronal brain sections with immunolabeling of microglia (Iba1, red) and staining for amyloid Aβ (6E10, green) in five-month-old 5XFAD mice kept on a standard diet or fed for 3 months with PLX3397 (290 mg/kg). Bottom panels: representative images of magnified regions of (1) hippocampus, (2) cortex, (3) thalamus and (4) amygdalar nucleus. The confocal acquisition was applied and a total z-plane of 30 μm was scanned across all regions and merged into single overlay images

**Fig. 3:**
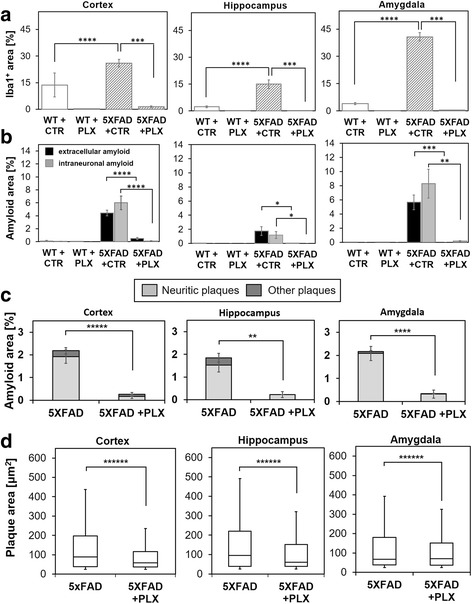
PLX3397 treatment reduces microglia, accumulation of intraneuronal amyloid and extracellular amyloid deposits in 5XFAD mice. **a** Area occupied by Iba1-positive microglia in the analyzed brains is increased by ~ 40%–90% in the regions of cortex, hippocampus and amygdala in 5XFAD mice compared to wild-type (*p* < 0.0001 denoted by ****). PLX3397 treatment eliminates ~ 99% of microglia in both wild-type and 5XFAD mice (*p* < 0.001 denoted by ***). Graph bars indicate mean ± SEM (*n* = 9/group). **b** Area fraction occupied by intraneuronal amyloid and extracellular amyloid deposits was quantified for the regions of cortex, hippocampus and amygdala independently. Long-term treatment with PLX3397 significantly prevented accumulation of the intraneuronal amyloid and nearly completely aborted formation of amyloid plaques in the cortex (*p* < 0.0001 denoted by ****). In the hippocampus, the accumulation of intraneuronal amyloid was significantly reduced (*p* < 0.024 denoted by *) as the formation of extracellular amyloid plaques (*p* < 0.05 denoted by *). In the amygdala region of the brains intraneuronal amyloid was reduced (*p* < 0.0002 denoted by ***) and formation of amyloid plaques was prevented (*p* < 0.003 denoted by **). Graph bars indicate mean ± SEM (*n* = 9/group). **c** Based on morphology and presence or absence of DAPI-positive core, extracellular amyloid was divided into neuritic or other types of plaques and quantified in brains. Treatment with PLX3397 significantly reduced area fraction occupied by neuritic plaques and other types of plaques (non-neuritic) in the cortex (*p* < 0.000005 denoted by *****), hippocampus (*p* < 0.005 denoted by **) and amygdala (*p* < 0.00001 denoted by ****). Graph bars indicate mean ± SEM (*n* = 12/group). **d** Size of amyloid plaques was quantified in three regions of brains. Treatment with PLX3397 significantly reduced the size of amyloid plaques in the cortex, hippocampus and amygdala (*p* < 0.000001, denoted by ******). Graph bars indicate mean ± SEM (*n* = 12/group and the total size of > 300 plaques/group were analyzed)

Two-month-old 5XFAD mice were selected for this study as they exhibit robust intraneuronal aggregated amyloid at 1.5 months, prior to neuritic plaque formation that begins at 2 months and is robust by 5 months, and display memory deficits starting by 4 months of age [18]. From earlier experiments we observed, that after at the age of 5 months non-treated 5XFAD mice showed both intraneuronal amyloid accumulation and excessive amyloid pathology (Fig. 1). We have observed in 5XFAD mice that the majority of extracellular amyloid deposits in the cortex, hippocampus and amygdala were a neuritic type of plaque with DAPI-positive center of the plaque surrounded by 6E10-positive rim and Iba1-positive cells and only a small fraction of all amyloid deposits were other than neuritic plaques (Fig. 1). During the 3 months treatment with PLX3397, no behavior or health problems were observed in mice, which is in line with previous findings by Kim Green and coworkers using short (21 days) and long term (2 months) PLX3397 treatment reported no such negative effects [9]. Surprisingly, we found that early treatment of two-month-old 5XFAD mice with PLX3397 for 3 months significantly reduced both the amount of intraneuronal amyloid and amyloid plaques when compared with non-treated 5XFAD mice (Fig. 2). Consistent with previous studies, treatment with PLX3397 significantly reduced (~ 70–80%) the number of Iba1-positive cells and reduced approximately 99% of the area fraction occupied by Iba1-positive cells in five-month-old 5XFAD mice (Fig. 3a) [9]. Compared with control animals, in 5XFAD mice treated with PLX3397, Iba1-positive cells appeared altered: they are much smaller and possess a few short and residual ramifications (Fig. 2). This is probably due to programmed apoptotic cell death triggered by inhibition of CSF1R by PLX3397 in Iba1-positive cells such as microglia and macrophages, which both express CSF1R [19].

PLX3397 treatment also led to a decrease in the accumulation of intraneuronal amyloid and extracellular amyloid deposits (Fig. 2). The decrease of intraneuronal amyloid and plaques is evident in all regions of the brain and is highly statistically significant (Fig. 3b, c). In this and our previous study [5] we have observed that the majority of extracellular amyloid deposits in the cortex, hippocampus and amygdala of 5XFAD mice are neuritic plaques with a DAPI-positive center of the plaque and a 6E10-positive rim of dystrophic neurites surrounded by Iba1-positive microglia. The treatment with PLX3397 removed ~ 90% of the neuritic plaques in all analyzed regions of the brains (Fig. 3c). The plaques that accumulate in PLX3397 treated brain are significantly smaller and more compact than the neuritic plaques in control mice (Fig. 3d) even though the plaques in PLX3397 treated mice are associated with the remaining microglia (Fig. 2). This indicates that the drug treatment not only decreases the number of amyloid plaques but also alters the process of plaque formation. Collectively, these data indicate that treatment of 5XFAD mice with PLX3397 leads to a reduction in both intraneuronal amyloid and neuritic plaque accumulation.

### PLX3397 administration improves behavioral phenotype

**Fig. 4:**
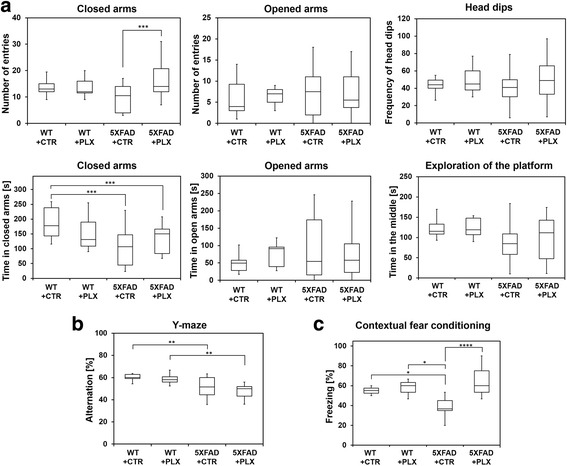
PLX3397 treatment improves behavioral phenotype. Contextual fear conditioning (**a**), Y-maze (**b**) and elevated plus maze (**c**) tests were performed. **a** In the contextual fear conditioning test, 5XFAD mice show significantly lower levels of contextual freezing than wild-type controls (*p* < 0.02, denoted by *). However, treatment with PLX3397 significantly reversed the impaired fear behavior to the level of a normal reconsolidation of the contextual fear memory (*p* < 0.00008 with Tukey correction test, denoted by ****). **b** The total number of arm entries was significantly changed in the group of 5XFAD mice regardless the treatment (*p* < 0.005 with Tukey correction test, denoted by **), indicating lower levels of motor and exploratory activity in the group of AD mice. **c** In the elevated plus maze test, although the number of entries into closed arms was increased in the 5XFAD mice treated with PLX3397 (*p* < 0.001 denoted by ***), the number of entries into opened arms was not significantly different among all four groups. Neither another behavioral parameter such as head dips, which is a complementary parameter to assess risky and or an exploratory behavior in the evaluation of anxiety, was not changed between groups. Whereas, 5XFAD mice spend less time in the closed arms (*p* < 0.0002, denoted by ***), regardless the treatment, there was no difference in the time spent in the opened arms or exploring the center zone of the platform. Box plots show median with interquartile lower and upper range and the minimum and maximum values. Total number of wild-type animals in the behavioral study was four animals (two males and two females) with drug and six animals without drug (three males and three females), respectively. The total number of animals in the behavioral study was 12 5XFAD animals (five males and seven females) with drug and 11 5XFAD animals without drug (six males and five females), respectively

Since PLX3397 treatment significantly reduces the amount of intraneuronal amyloid and neuritic plaques accumulation, we examined whether this effect on amyloid pathology would result in an improvement in the behavioral phenotype of 5XFAD mice. Thus, mice were tested in two hippocampal tests, the Y-maze and contextual fear condition, allowing us to determine whether PLX3397 treatment mitigate the spatial and emotional memory deficits observed in these mice. Our data demonstrate that PLX3397 treatment reverses the emotional and contextual memory as 5XFAD mice showed an improvement of a fear-associated memory when treated with PLX3397 (Fig. 4a). However, no changes were observed in spatial hippocampal memory (Fig. 4b). It is not clear why some measures of hippocampal function respond to drug treatment and others do not, but it seems to indicate that some measures of AD-related behavioral deficits can be dissociated by drug treatment. In addition, the animals did not show any differences in exploratory and anxiety-like behavior in an elevated plus maze test, as no differences in the numbers of entries in the open versus closed arms, the overall exploration of the middle part of the platform and manifestation of a risky behavior is observed between groups (Fig. 4c). Taken together, our findings implicate a strong relationship between amyloid pathology, microglia and amygdala and hippocampus-related behaviors in 5XFAD mice that are reversed by a treatment with CSF1R inhibitor and microglia reduction.

### Treatment with PLX3397 lowers the levels of pre-fibrillar oligomers in plasma and fibrillar amyloid in the brain

**Fig. 5:**
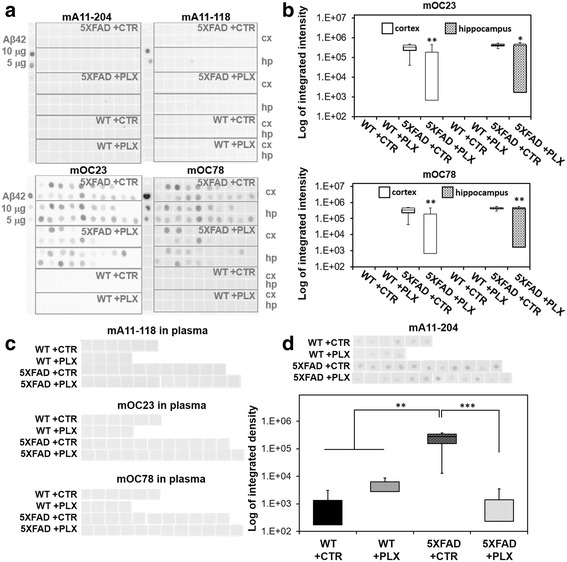
Treatment with PLX3397 reduces the level of fibrillar amyloid in the total brain lysates and pre-fibrillar oligomers in the blood plasma of 5XFAD mice. **a** An equal amount (6 μg) of total lysate from the cortex (cx) or hippocampus (hp) of each group of mice (male and female) were spotted on the membrane and incubated with antibodies specifically detecting pre-fibrillar oligomers (mA11) or fibrillar aggregates (mOC). 5 μM of oligomerized for 3 days Aβ42 was spotted as a control for antibodies to confirm the ability of the antibodies to distinguish between oligomeric vs. fibrillar conformation. Additionally, 5/10 μg of rabbit IgG were spotted as a positive control for the secondary anti-rabbit antibody. **b** The dot blot analyses were quantified for the mOC23 and mOC78. Whereas wild-type brains were free of amyloid, the fibrillar soluble amyloid was increased in the cortex and hippocampal lysates. However, long-term treatment with PLX3397 (290 mg/kg) decreased significantly the amount of soluble fibrillar mOC23 amyloid in the cortex (*p* < 0.004 with Tukey test, denoted by **) and in the hippocampus (*p* < 0.05 with Tukey test, denoted by *) and soluble fibrillar mOC78 amyloid in the cortex (*p* < 0.0018 with Tukey test, denoted by **) and in the hippocampus (*p* < 0.0012, denoted by **) when compared with the control-chow treated 5XFAD mice. **c** An equal amount of blood plasma (1 μg) of each group (male and female) was spotted on the membrane and incubated as in (**a**). Neither the antibodies against fibrillar mOC23, mOC78 nor mA11–118 against pre-prefibrillar oligomers detected a soluble amyloid in blood plasma. However, antibody mA11–204 has detected pre-fibrillar amyloid in the blood plasma of analyzed cohorts. **d** Quantification of the dot blot for mA11–204 presence in blood plasma. Some endogenous pre-fibrillar amyloid was detected in the wild-type animals, however, the amount of mA11–204-positive amyloid increased significantly in the plasma of 5XFAD animals (*p* < 0.0011 with Tukey test, denoted by **). Long-term administration of PLX3397 in young 5XFAD decreased the amount of pre-fibrillar oligomers (mA11–204) in blood plasma (*p* < 0.00082 with Tukey test, denoted by ***). A-D each assay was performed once for each individual animal due to non-significant inter-assay variability

Based on our observation that PLX3397 treatment inhibited intraneuronal amyloid accumulation and neuritic plaque deposition, we were interested in determining how the levels of pre-fibrillar oligomers and soluble fibrillar amyloid oligomers change in response to treatment. In total brain lysates, pre-fibrillar oligomers were not detected in any of the cohorts, however, the level of soluble fibrillar amyloid, detected by monoclonal antibodies mOC23 and mOC78, is significantly reduced by drug treatment (Fig. 5a-b). Consistent with the increase in amyloid plaques observed by immunofluorescence, we found that the level of soluble fibrillar amyloid was increased in 5XFAD mice in both cortex and hippocampus. The levels of fibrillar amyloid were significantly reduced by the treatment with PLX3397 (Fig. 5b).

In contrast to the results obtained with brain lysates, we have shown that pre-fibrillar oligomers, but not fibrillar oligomers were detected in plasma (Fig. 5c). Interestingly, the level of mA11–204-positive amyloid oligomers was significantly increased in the 5XFAD mice when compared with the wild-type littermates, whereas treatment with PLX3397 significantly decreased the levels of those amyloid oligomers (Fig. 5d). Together, these data support the findings that treatment with PLX3397 reduces the level of soluble fibrillar oligomers in the brain and the level of pre-fibrillar, toxic oligomers in plasma.

## Discussion

A rapidly growing body of evidence indicates that microglia play an important role in brain homeostasis, neuroinflammation and neurodegenerative disorders such as AD (reviewed in [20, 21]). We and others have suggested that intraneuronal amyloid might play a crucial role in the pathophysiology of AD, especially as neuritic plaques are believed to originate from dying neurons with intraneuronal amyloid [5, 22–24]. Here we demonstrate early long-term administration of PLX3397 dramatically reduces microglia levels and inhibits both intraneuronal amyloid and neuritic plaque accumulation and ameliorates emotional and contextual memory deficits. Thus, early long-term administration seems to be crucial to significantly reduce amyloid pathology, since other investigators have reported that PLX3397 and other CSF1R inhibitor-induced ablation of microglia does not cause a reduction in amyloid plaque load when treatment is initiated at later times after robust amyloid plaque deposition in 5XFAD mice [10]. The simplest interpretation for this difference is that CSF1R inhibition and microglia ablation prevent the formation of neuritic plaques, but do not have an impact after plaques have already formed. The fact that intraneuronal amyloid precedes neuritic plaque formation and both types of deposits are dramatically reduced by PLX3397 treatment suggests that intraneuronal amyloid deposits and neuritic plaque amyloid are linked in a causal series. If instead intraneuronal amyloid accumulation and neuritic plaque deposition were coincidental but independent events, it would be unlikely that the drug would inhibit both pathways independently because the drug has been shown to have no effect on Aβ production in 5XFAD mice [10]. While inhibition of neuritic plaque formation could be easily explained as a consequence of the necessity of microglial phagocytosis for plaque formation, the effect of CSF1R inhibition and microglia ablation on intraneuronal amyloid is more surprising and puzzling. It implies that either microglial signaling to neurons or CSF1R signaling on neurons is necessary for intraneuronal amyloid accumulation. Using lineage-tracing experiments, Wyss-Coray and coworkers reported that a small number of neurons in the hippocampus and cortex express CSF1R under physiological conditions [25]. Thus, it is possible that CSF1R signaling in neurons is responsible for promoting the accumulation of intraneuronal amyloid. An alternate explanation is that microglia might regulate the accumulation of intraneuronal amyloid by producing inflammatory cytokines and chemokines that promote intraneuronal amyloid accumulation [26, 27]. Both hypotheses require further investigation.

If intraneuronal amyloid leads to neuronal death and initiates neuritic plaques, then the inhibition of CSF1R signaling or CSF1R-dependent microglial signaling would be protective for AD according to this model. This is the opposite of the more widely held view that CSF1R signaling is neuroprotective. A study by Luo and coworkers reported that CSF1 ameliorated memory deficits in the hAPP mouse model and CSF1 and IL34 administration strongly reduced excitotoxic neuronal loss, while deletion of CSF1R in neurons exacerbated excitotoxic neuronal death [25]. Although the reason for this difference is not yet clear, the simplest interpretations is that it is due to the differences between the specific genetic ablation in a subset of neurons while pharmacological inhibition hits more targets in more cell types. It may also suggest that amyloid pathogenesis is regulated in a different CSF1R-dependent mechanism than excitotoxicity. In contrast to the neuroprotective functions, CSF1R signaling during amyloid pathogenesis might instead activate pro-inflammatory processes. A similar pro-inflammatory function of CSF1R signaling has been described in an animal model of arthritis, where antibody blockade of CSF1R signaling significantly reduced the inflammatory response and ameliorated disease-related symptoms [28]. Our results also indicate that microglia are closely associated with Aβ deposition because the remaining plaques after drug treatment are still surrounded by the remaining microglia, even though both are strongly reduced in number. The remaining plaques in drug-treated animals are smaller, which indicates that the process of plaque formation is also altered by drug treatment. Whatever the explanation is for the role of CSF1R signaling on intraneuronal amyloid accumulation, the fact that neuritic plaque formation is prevented suggests that inhibition of CSF1R signaling may be a preventative approach, or therapeutic when applied at the very earliest stages of amyloid deposition during AD.

Amyloid oligomers are widely believed to be a primary toxic species of Aβ. Amyloid oligomers are structurally polymorphic and can be classified as prefibrillar oligomers (A11+/OC-) or fibrillar (A11-/OC+) oligomers on the basis of their differential immunoreactivity with conformation-dependent antibodies [29–32]. We present a novel finding that treatment with PLX3397 lead to a significant reduction of mA11–204 positive prefibrillar oligomers in plasma of 5XFAD mice. This result may reflect a neuroprotective activity of drug treatment as studies have shown that prefibrillar oligomers are neurotoxic [33]. We have detected prefibrillar oligomers only in plasma, but not in brain lysates of 5XFAD mice, perhaps due to their preferential secretion or transport into the bloodstream. We have also observed low levels of prefibrilar oligomers, detected by A11–204 monoclonal antibody, in wilde-type mice. The presence of A11–204 pre-fibrilar oligomers might represent kind of “background noise” since oligomer-specific A11–204 also recognizes soluble oligomers from various proteins, such as α-synuclein, islet amyloid polypeptide, polyglutamine (PolyQ), lysozyme, human insulin and prion peptide [8, 29]. Importantly, treatment with PLX3397 reduced level of toxic pre-fibrillar A11-positive oligomers to the level of “background noise” of healthy individuals.

Finally, our behavioral studies showed that long-term administration of PLX3397 in young 5XFAD mice improved associative learning (contextual fear conditioning), which had also been reported for shorter administration in older ten-month-old 5XFAD mice [10]. The contextual fear conditioning test investigates the involvement of both amygdala and hippocampus [34], in which atrophy can be detected in the earliest clinical stages of AD [35]. Other behavioral tests on spatial memory (Y-maze test) or anxiety (elevated plus-maze test) showed only a beneficial trend, but none of them was statistically significant, suggesting that a longer treatment may be necessary to recover these behavioral tests. Taken together, we demonstrated that early long-term treatment with the CSF1R inhibitor, PLX3397 significantly reduced intraneuronal amyloid, neuritic plaque formation, a reduced amount of toxic prefibrillar oligomers and improved cognitive function in particular associative learning in the contextual fear conditioning of 5XFAD mice. These data show that CSF1R signaling, either through microglia or directly on neurons profoundly contributes to amyloid pathogenesis.

## Conclusions

CSF1R signaling significantly contributes to the accumulation of intraneuronal amyloid and formation of neuritic plaques in 5XFAD model. Long-term treatment of young 5XFAD mice with PLX3397 lead to an ablation of microglia, a reduction of intraneuronal amyloid and neuritic plaque accumulation. Moreover, the level of soluble fibrillar amyloid in the brain and pre-fibrillar oligomers in blood plasma was significantly reduced. Overall, treatment with PLX3397 improved hippocampal contextual memory. These results suggest that inhibition of CSF1R signaling may be a preventative approach, or therapeutic when applied at the very earliest stages of amyloid deposition during AD.

## Abbreviations

5XFAD: Transgenic mouse model carrying five familial Alzheimer’s disease mutations (B6SJL-Tg(APPSwFlLon,PSEN1*M146 L*L286 V)6799Vas/Mmjax)
AD: Alzheimer’s disease
APP: Amyloid precursor protein
Aβ: Amyloid β
CSF1R: Colony stimulation factor 1 receptor
FAD: Familial Alzheimer’s disease
Iba1: Ionized calcium binding adaptor molecule 1
IL34: Interleukin 34
NeuN: Neuronal nuclear protein
NFTs: Neurofibrillary tangles
PS: Presenilin
